# An automated approach to prepare tissue-derived spatially barcoded RNA-sequencing libraries

**DOI:** 10.1038/srep37137

**Published:** 2016-11-16

**Authors:** Anders Jemt, Fredrik Salmén, Anna Lundmark, Annelie Mollbrink, José Fernández Navarro, Patrik L. Ståhl, Tülay Yucel-Lindberg, Joakim Lundeberg

**Affiliations:** 1 Science for Life Laboratory, Department of Gene Technology, Royal Institute of Technology, SE-106 91, Stockholm, Sweden; 2 Department of Dental Medicine, Division of Periodontology, Karolinska Institutet, SE-141 04, Huddinge, Sweden; 3 Department of Cell and Molecular Biology, Karolinska Institutet, SE-171 77 Stockholm, Sweden

## Abstract

Sequencing the nucleic acid content of individual cells or specific biological samples is becoming increasingly common. This drives the need for robust, scalable and automated library preparation protocols. Furthermore, an increased understanding of tissue heterogeneity has lead to the development of several unique sequencing protocols that aim to retain or infer spatial context. In this study, a protocol for retaining spatial information of transcripts has been adapted to run on a robotic workstation. The method spatial transcriptomics is evaluated in terms of robustness and variability through the preparation of reference RNA, as well as through preparation and sequencing of six replicate sections of a gingival tissue biopsy from a patient with periodontitis. The results are reduced technical variability between replicates and a higher throughput, processing four times more samples with less than a third of the hands on time, compared to the standard protocol.

RNA sequencing (RNA-seq) is becoming one of the standard tools for studying the dynamic life of tissues and cells^1^. Increased sample throughput and clever barcoding strategies allow for several sample libraries to be sequenced in a single instrument run^2^,^3^,^4^,^5^,^6^. Aside from increasing sample throughput, barcoding also allows researchers to account for amplification biases, which can arise when amplifying a small number of starting molecules^7^,^8^.

With improved sequencing capacity, demand for increased sample preparation throughput has risen. This has been achieved by setting up library preparation protocols on robotic workstations^9^,^10^,^11^,^12^,^13^,^14^,^15^. It has previously been shown that a 12-channel liquid handling robot can increase sample preparation throughput by up to six times^9^. Robots with 96-channels have increased throughput by up to 16 times^10^. Aside from increased throughput, automation offers advantages in robustness and cost, as well as minimized risks for cross contamination and human error^16^,^17^.

There has recently been an increased interest in developing methods to add a layer of spatial information to RNA-seq experiments, allowing new insights into tissue heterogeneity^18^,^19^,^20^,^21^,^22^. One such approach is spatial transcriptomics^23^, which utilizes a glass slide arrayed with barcoded cDNA primers. Thin tissue sections are placed on the array, stained and imaged, before the RNA molecules are captured and made into cDNA directly underneath the cells. By using a modified version of the CEL-seq protocol^24^ the barcoded cDNA is prepared into sequencing libraries. Overlaying the resulting spatial RNA-seq data onto a high-resolution tissue image provides unique possibilities for subsequent *in situ* analysis. Here we describe a protocol for automating the generation of these barcoded sequencing libraries, resulting in an increased robustness and minimal hands on time.

## Results

### Automation of spatial transcriptomics

**Figure 1:**
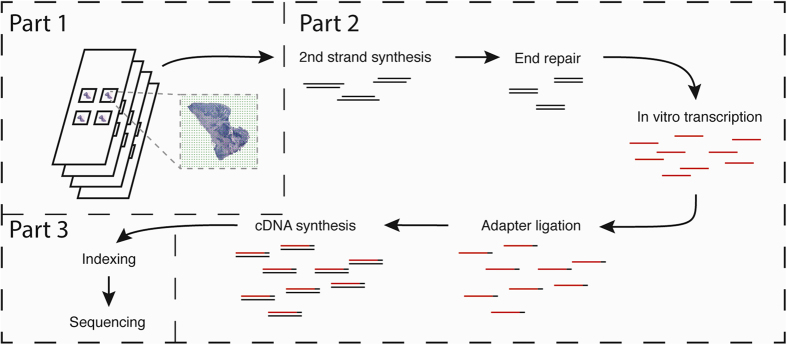
Overview of library preparation steps. The library preparation can be divided in three parts in which the second part is performed by the robotic workstation. In part one, fresh frozen tissue sections are mounted on a barcoded array, and cDNA is synthesized from the mRNA in the tissue section. In part two, cDNA is transferred from the surface of the chip to the robot. The robot performs second strand synthesis and end repair, followed by *in vitro* transcription, adapter ligation and cDNA synthesis. Each step is accompanied by a reaction clean-up using paramagnetic carboxylic acid beads. Part three consists of sample indexing by PCR and, following clean-up, the sample is ready for sequencing.

The protocol is an adaptation of the spatial transcriptomics method described previously^23^ and consists of three parts (Fig. 1). The first part takes place on a custom microarray glass slide with designated sub-arrays. Oligonucleotides have been printed in each sub-array and each spot (100 μm in diameter) contains approximately 200 million probes. The probes structure, starting from the surface, contains a uracil cleavage region, a T7 amplification handle, a partial sequencing handle, a cluster specific sequence (spatial barcode), a semi-randomized unique molecular identifier (UMI) and an oligo-dT mRNA capture sequence which functions as a primer for cDNA synthesis. Sections of frozen tissue are placed on the array and fixed using formalin. The tissue sections are then stained with Haematoxylin and Eosin and imaged. The tissue is permeabilized and the RNA is reversely transcribed into barcoded cDNA. The tissue is removed and the RNA/cDNA is enzymatically cleaved from the array.

The second part starts with the released cDNA, which is transferred to the robotic workstation where it undergoes second strand synthesis, end repair and *in vitro* transcription, with a reaction clean-up after each step. Following transcription, sequencing adaptors are ligated to the RNA, and another round of cDNA synthesis is performed. Each step in the process is followed by a reaction clean-up step.

The third and final part of the library preparation starts with indexing of the sequencing samples by PCR to allow for multiplexing of libraries from different tissue sections. The samples are then purified on a robotic workstation, using PEG precipitation on carboxylic acid beads^9^, in order to yield high quality spatial sequencing libraries.

Following this protocol allows for up to eight samples to be prepared simultaneously, after the on-array reactions. The total time from cleaved cDNA to indexing samples is 24 hours and 40 minutes, including hands on time of approximately two hours. Using pre-aliquoted reagents can reduce the hands on time to approximately 20 minutes. The manual protocol allows for the preparation of four samples in parallel and takes a comparable amount of total time to the automated protocol, but requires up to six hours of hands-on time. Furthermore, the automated protocol can be operated in a mode that allows up to 16 samples to be prepared in parallel with less than 10% increase in total time (Table 1).

### Reproducibility

**Figure 2:**
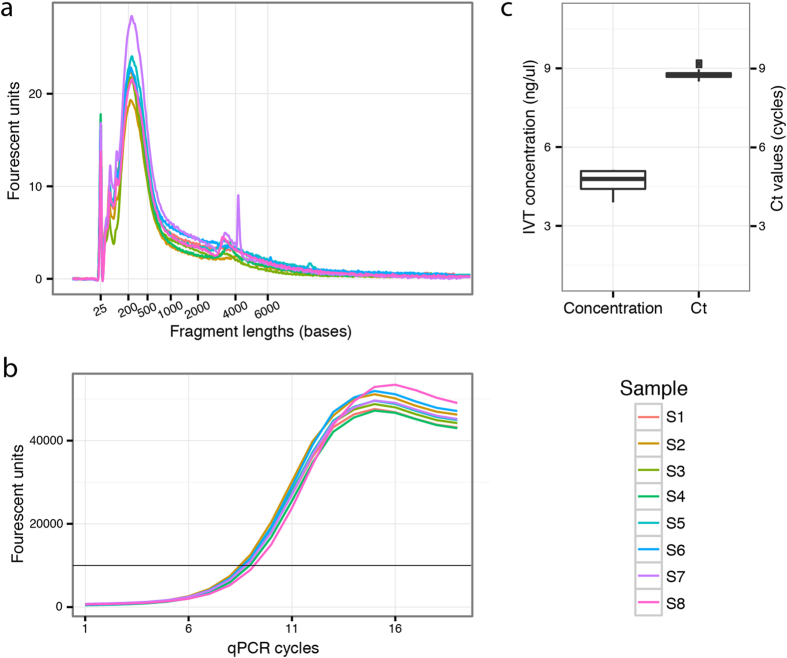
Quantitative evaluation of libraries prepared from total reference RNA. Sample-to-sample variation was investigated by small red triangles assessing the libraries at two points during the library preparation process. (**a**) The first evaluation is performed after *in vitro* transcription and checks the library concentrations and fragment lengths using a Bioanalyzer (Agilent). (**b**) After reverse transcription, a quantitative PCR (qPCR) was carried out to determine the suitable number of PCR cycles when indexing the finished libraries. The black vertical line marks the signal threshold at which point the cycle threshold (Ct) values were obtained. The spread of Ct values is illustrated in the boxplot (**c**), which also shows the variation in sample concentration as measured by the Bioanalyzer after *in vitro* transcription.

The technical variability of the protocol was evaluated by processing 8 identical replicates in parallel, originating from the same total RNA batch. Any sample-to-sample variation between the replicates could then be attributed to the robot. The quality of the libraries was evaluated at two points during the process, first through analysing the fragment lengths and library concentration after *in vitro* transcription, and then by analysing the library amplifiability after the final cDNA synthesis. The sample-to-sample variation is illustrated in Fig. 2. The average concentration after *in vitro* transcription was calculated as 4.8 ng/μl with a standard deviation of 0.4 ng/μl (Fig. 2a,c). The samples were further evaluated by quantitative PCR (qPCR) after the final cDNA synthesis (Fig. 2b,c), showing very small variations in cycle threshold (Ct) values between samples.

### Comparing automated and manual preparations

**Figure 3:**
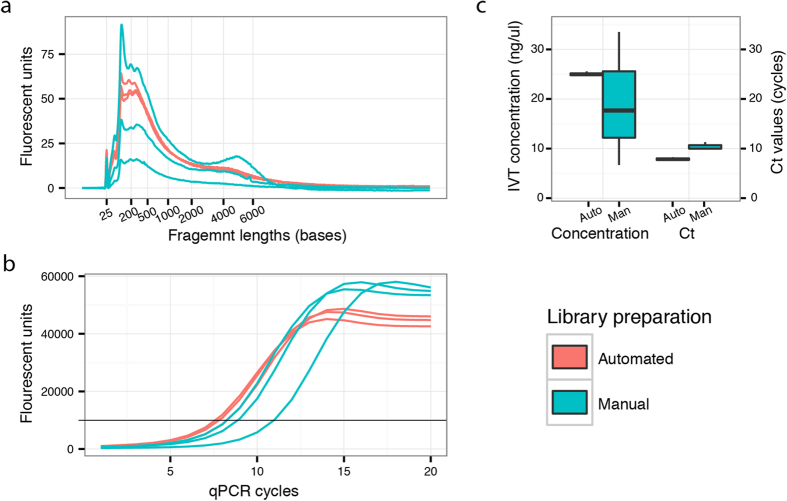
Quantitative evaluation of libraries prepared from oral gingival tissue. The quality of the libraries generated from gingival tissue was assessed after *in vitro* transcription and again after final cDNA synthesis. (**a**) Bioanalyzer (Agilent) trace showing the fragment size distributions for the six libraries after amplification by *in vitro* transcription. The traces from the automated protocol are shown in red while the traces from the manual protocol are shown in blue. The traces were also used to estimate the concentrations of the samples, which are illustrated in the left part of the boxplot (**c**). After the final cDNA synthesis the libraries were evaluated by quantitative PCR (qPCR) (**b**). The black vertical line marks the signal threshold at which point the cycle threshold (Ct) values were noted. The Ct values for the libraries as obtained from the qPCR are plotted in the right part of the boxplot (**c**).

The automated protocol was benchmarked against the manual procedure by preparing and sequencing six libraries, which were obtained from adjacent sections in the same oral gingival tissue biopsy. Three samples were prepared using the manual procedure and three were prepared using the automated protocol. The libraries were evaluated during the preparation process using Bioanalyzer (Agilent) and qPCR (Fig. 3). The manually prepared libraries exhibited greater between sample variation than the libraries prepared using the automated protocol. Concentration measurements after *in vitro* transcription had a coefficient of variation (CV) of 57% for the manual samples and 1.5% for the automated samples.

The libraries were sequenced on a NextSeq (Illumina) instrument, generating an average of 194 million reads per sample. For reasons of comparability the samples were down-sampled to the library with the least number of reads (171 million reads). The reads from the automated libraries were of very consistent quality with on average 143 million reads (CV: 0.11%) remaining after quality trimming the down-sampled reads, while the manually prepared libraries had on average 127 million reads remaining (CV: 7.81%). After mapping to the human genome (GRCh38) and annotating using only polyadenylated transcripts, the number of unique transcripts could be determined. On average 4.38 million unique transcripts could be identified in the automated libraries and 3.39 million in the manual libraries, with a CV of 8.54% and 10.64% respectively. The results from the sequencing are summarized in Supplementary Table 1.

**Figure 4:**
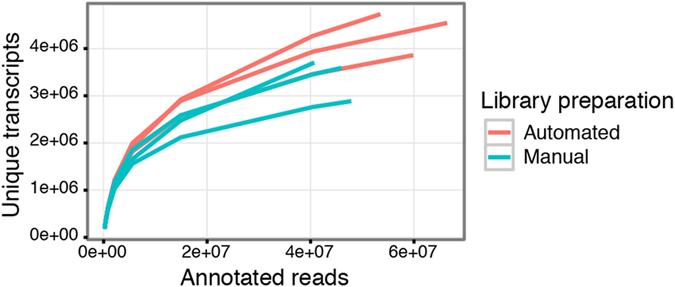
Saturation curves for the sequenced libraries. The annotated reads were down-sampled to several pre-determined sequencing depths and for each point the number of unique transcripts were counted. The curves show a similar level of saturation, with the automated libraries having slightly more unique molecules at the analysed sequence depth. All libraries had been down sampled to the same amount of raw reads prior to mapping and annotation.

Down-sampling the annotated reads and counting the number of unique transcripts at each sampling point made it possible to estimate the diversity of each library at the down-sampled sequencing depth. This also allowed for insights into whether the libraries had been sequenced to saturation. Although the results were similar for all libraries, it was apparent that the automated libraries, on average, exhibited a higher diversity than their manual counterparts (Fig. 4).

**Figure 5:**
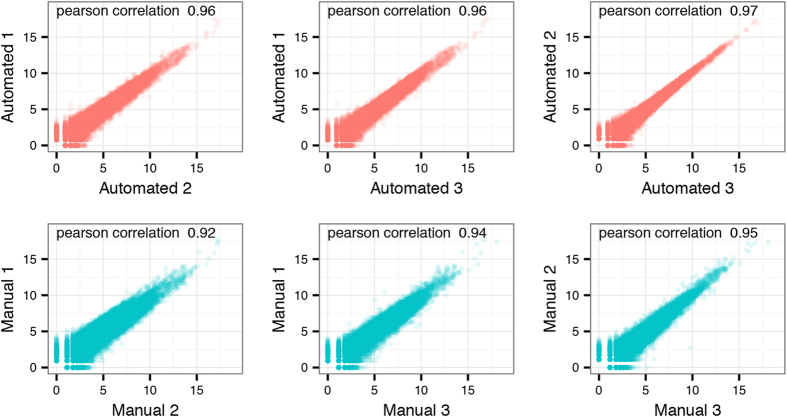
Correlations within the replicate groups. Pairwise comparison of the log2 normalized gene counts for the automated and manually prepared libraries. The automated libraries are plotted in the top part of the figure (red), while the manual libraries are in the lower part (blue). Each plot includes the Pearson correlation score for the pair.

The normalized gene counts from each library were log2-transformed and the variation between replicates was compared. Average Pearson correlation scores were slightly higher in the group of libraries prepared using the automated protocol (0.96) when compared to the manually prepared libraries (0.94) (Fig. 5).

### Variation on the spatial level

**Figure 6:**
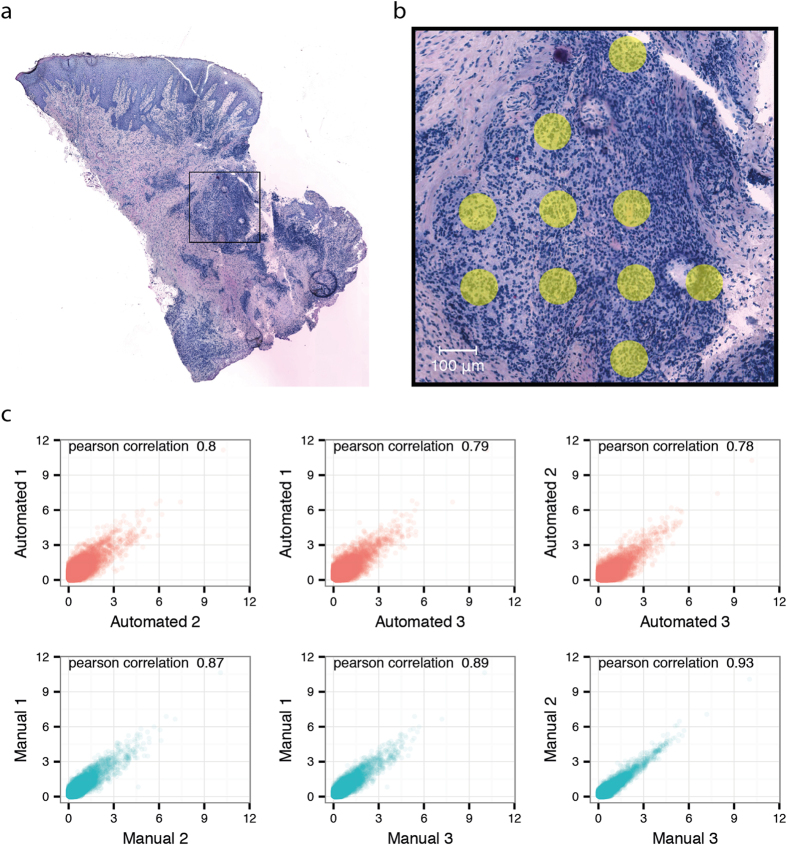
Overview of tissue section and selected spots for comparison of inflamed regions. Bright field image of one of the gingival tissue sections stained with haematoxylin and eosin. The selected inflamed area has been marked with a black rectangle (**a**). Ten spots within this area were selected in all the six tissue sections (**b**). For each section, the transcripts mapping back to these spots were added together based on gene annotation and averaged over the ten spots. The log2 normalized gene counts of this average spot from each region were compared between the tissue sections. Upper panel show the correlations between inflamed regions of the automated samples. The lower panel plots shows the correlations within the manually prepared samples (**c**).

The sample variation was also interrogated on the spatial level by investigating gene expression averaged over ten spots in an inflamed region (Fig. 6a,b). Due to the tissue sections being taken consecutively from the same block, the same inflamed region could be identified and selected in all the sections. The level of correlation within samples prepared automatically and samples prepared manually was calculated and plotted (Fig. 6c). When compared to an analysis of the samples in bulk, the overall correlation values for the spatially selected areas were lower, with a maximum of 0.93 and a minimum of 0.78. The correlation values between the manually prepared libraries were lower (average correlation scores 0.79) compared to the automated libraries (average correlation score 0.90), indicating higher technical variability for manually prepared libraries. The number of genes and unique transcripts inside the selected inflamed regions were both higher and exhibited lower variation between replicates for the automated libraries compared to the manual libraries (Supplementary Figure 1).

## Discussion

We have automated a complex library preparation protocol for transcriptome sequencing. The modular nature of the protocol makes it easy to remove and add steps as the library preparation procedure is further developed. The current workstation has a maximum capacity of 32 samples in parallel. Further parallelization to 96 and 384 samples would require a transfer of the protocol to a higher capacity robotic unit.

Even with highly skilled personnel, sectioning fresh frozen tissue and placing sections on the microscopic glass microarray is a delicate process. The difficulty of sectioning fresh frozen tissue varies between tissue types and with environmental conditions, such as humidity. This variability between tissue sections makes them suboptimal for demonstrating the reproducibility of the automated method. Thus, in order to assess whether the robot introduces any variability between samples, cDNA from Human reference RNA prepared from a single first strand synthesis reaction in solution was used as input for the robot in the initial experiment. Using reference RNA guarantees the same input for all the libraries and any difference between the samples after the library preparation is finished can then be accredited to the library preparation process. The libraries were evaluated with respect to size and concentration at an intermediate quality control step as well as with qPCR after the final cDNA synthesis. Generally there were very small variations between samples, leading us to conclude that the script regulating the robots movements and pipetting was sound and robust and would have very little impact on any future observed variation between samples.

The automated protocol was compared to manual preparation using six adjacent sections from a gingival tissue biopsy obtained from a patient with periodontitis. The samples were evaluated at the same steps as the reference RNA-derived samples. The variation between the manually prepared samples is striking considering they were prepared at the same time by the same person, and shows the amount of variation that can be introduced in manual preparations. The samples were sequenced on the NextSeq (Illumina), yielding an average of 194 million read pairs per sample, which were down-sampled to 170 million read pairs. The number of unique transcripts for each sample was then identified, and the correlation between replicates calculated. We acknowledge that having individual tissue sections as sample material makes it hard to draw absolute conclusions regarding robustness. Nevertheless, more unique transcripts and genes were detected in the automatically prepared libraries when compared to the manually prepared libraries, accounting for the same number of input reads, and the correlation scores indicated that the automated protocol introduced less technical variation. Further, the fraction of unique transcripts recovered is consistent with the fraction previously reported^23^.

We analysed gene expression correlation between samples both for the whole tissue sections and for an isolated region with infiltrated inflammatory cells. Using whole tissue sections we observed a Pearson correlation ranging between 0.96 and 0.97 for the automated libraries, and correlation values ranging between 0.92 and 0.95 for the manually prepared libraries. When using only an inflamed region for analysis, correlation values of between 0.87 and 0.93 were observed for the automated libraries, and correlation values of between 0.78 and 0.80 for the manual libraries. The slightly lower correlation values for the inflamed region compared to bulk data can most likely be attributed to a smaller sample size at which an increased dropout rate is to be expected^25^.

To conclude, we have automated a library preparation protocol for spatial transcriptomics, a method for two-dimensional tissue transcriptome sequencing that retains the spatial information in the tissue section for each transcript. The automated protocol has been proven to be more robust than the standard manual procedure, as demonstrated by the preparation and sequencing of gingival tissue replicates. Aside from minimizing human errors, this novel automation allows for more a cost-efficient protocol through increased throughput and reduced hands-on time.

## Methods

The manual protocol was adapted for the Magnatrix 8000 + (Nordiag), an eight channel robotic workstation capable of running custom made scripts for in-tip magnetic bead separations. The instrument features two Peltier units (4–95 °C), one of which was used for the enzymatic reactions and the other for the storage of heat sensitive reagents. Adaptions included the removal of a volume reduction step prior to the *in vitro* transcription that utilized a vacuum concentrator. In order to omit this step but still reach the concentrations of the manual protocol the first reaction clean-up with Agencourt RNAClean XP (Beckman Coulter) beads was modified. Instead of eluting the product in 20 μl water, the samples were eluted in a 12 μl 40 mM nucleotide solution. Since the workstation was not capable of sealing reaction plates, an oil solution (Vapor-Lock, Qiagen), was used to cover the reactions and prevent evaporation loss and contamination. Details about the library preparation steps and oligonucleotide sequences can be found in the supplementary information of Ståhl, Salmén *et al.*^23^. Instructions on how to install the protocols on a Magnatrix 8000 + system as well as considerations when transferring the protocol to other laboratories and liquid handling systems can be found in the supplementary information.

### Reproducibility library

Eight sequencing libraries were created from Human Reference RNA (Agilent) in order to assess the variation between samples in a robot run. 18 μg of RNA was fragmented at 94 °C for 3 minutes using the NEBNext® Magnesium RNA Fragmentation Module (New England Biolabs) and a MinElute Cleanup kit (Qiagen) was used for reaction clean-up. Size distribution was assessed using a Bioanalyzer (Agilent) and concentration was measured using a Qubit (Thermo Fisher Scientific). cDNA was created by first incubating 5 μg of fragmented RNA in a 28 μl solution containing dNTP (Thermo Fisher Scientific) containing a custom biotinylated oligo dT primer (integrated DNA Technologies) for 5 min at 65 °C. A 12 μl reverse transcriptase mix was added to the sample to give a final concentration of 1 x First Strand Buffer (Thermo Fisher Scientific), 0.5 mM dNTP, 2 μM oligo dT primer, 5 mM DTT (Thermo Fisher Scientific), 2 U/μl RNaseOut (Thermo Fisher Scientific), 20 U/μl Superscript III (Thermo Fisher Scientific). The reaction was incubated at 50 °C for 60 minutes, followed by 15 minutes at 70 °C, before being placed on ice. 40 μl of Dynabeads MyOne Streptavidin T1 beads (Thermo Fisher Scientific) were washed according to the manufacturer’s protocol and the sample was added to the beads. After a 10 minute incubation in room temperature the beads were washed in 2x SSC (Sigma-Aldrich) at 50 °C for 10 minutes followed by 0.2x SSC for 1 minute in room temperature and finally 0.1x SSC in room temperature for 1 minute. A 40 μl release mix (1x Second Strand Buffer (Thermo Fisher Scientific), 0.2 μg/μl BSA (New England Biolabs), 0.5 mM dNTP (Thermo Fisher Scientific) and 0.1 U/μl USER Enzyme (New England Biolabs)) was added to the beads and the mix was incubated at 37 °C for 60 minutes. The beads were separated from the liquid and discarded. Eight aliquots, corresponding to 100 ng of starting RNA, were taken from the cDNA reaction and diluted in 1x Second Strand Buffer (Thermo Fisher), 0.2 μg/μl BSA (New England Biolabs), 0.5 mM dNTP (Thermo Fisher Scientific) to a final volume of 65 μl each. This was used as input for the variance test. Average fragment length and quantity after *in vitro* transcription using were assessed using an RNA Pico Kit on a 2100 Bioanalyzer (Agilent) according to the manufacturer’s protocol. After final cDNA synthesis qPCR was carried out to further investigate sample variability. The qPCR was performed using primers targeting the adaptors^23^ that had been ligated to the fragments during the earlier stages of the library preparations. The cycle threshold (Ct) values obtained from the qPCR is proportional to the amount of available starting molecule and thus a tight distribution of Ct values implies a similar number of starting molecules.

### Gingival tissue library

A gingival tissue biopsy was obtained from a patient with the chronic inflammatory disease periodontitis under ethical approval from the Regional Ethics Board in Stockholm (Dnr: 2008/1935–31/3) and with written informed consent from the participant. All experiments were carried out in accordance with the approved guidelines.

The biopsy was snap-frozen, embedded in OCT and sectioned serially at a thickness of 10 μm on a cryostat. Six sections were selected and mounted on a barcoded array for library preparation. Libraries for three of the sections were prepared on the robotic workstation while the other three were prepared manually. The libraries were assessed at the same intermediate steps as the reproducibility libraries. After the final cDNA synthesis the libraries were amplified by PCR using Illumina compatible indexing primers and sequenced on a NextSeq 500 to a minimum depth of 170 million reads. The NextSeq was programmed to generate 36 base pair forward and 121 base pair reverse paired end reads. The shorter forward read contains a barcode that can be used to map the read back to the spatial position of the microarray as well as an UMI to be able to account for PCR duplicates.

The fastq files were down-sampled to the library with the least amount of reads using Seqtk (https://github.com/lh3/seqtk). The reverse reads were processed with a BWA style trimming script to remove low quality sequences. Ribosomal reads were removed by aligning the reverse reads to ribosomal sequences using STAR^26^. The remaining reads were mapped to the human genome (GRCh38) using STAR. The number of reads that aligned to each gene was counted using HTSeq-count^27^ and an annotation file that contained only poly-adenylated transcripts. Reads that aligned to an annotated region were then filtered using the spatial barcode from the forward read. Duplicate reads with the same spatial barcode were then removed using the UMI sequence on the forward read and a file containing the gene count data for each spatial barcode was produced. Saturation curves were calculated by down sampling the annotated reads to predefined sequencing depths and for each point calculating the number of unique transcripts.

For bulk analysis, the counts for each gene were summed over all the spatial barcodes in the sample. For comparison of expression in the inflamed region in each of the sections, ten spots aligning directly underneath the inflamed region were selected and the count data from this region was averaged. The count data for the inflammatory regions and the whole tissue section were normalized using the DESeq2 package^28^, a pseudo count of one was added followed by log2 transformation. Pearson correlation scores were then calculated for each pairwise comparison within each sample group and correlation plots were drawn. The variation in number of recovered genes and unique transcripts between the spots in each of the inflamed regions were compared (Supplementary Figure 1).

## Additional Information

**How to cite this article**: Jemt, A. *et al.* An automated approach to prepare tissue-derived spatially barcoded RNA-sequencing libraries. *Sci. Rep.* **6**, 37137; doi: 10.1038/srep37137 (2016).

**Publisher’s note**: Springer Nature remains neutral with regard to jurisdictional claims in published maps and institutional affiliations.

## Supplementary Material

Supplementary Information

## Figures and Tables

**Table 1:** Time comparison of manual and automated protocol.

Steps	Manual, 1–4 samples		Automated, 8 samples		Automated, 16 samples	
	Hands on	Total	Hands on	Total	Hands on	Total
2nd strand synthesis	30 min	3 h 10 min	1 h	3 h 10 min	1 h	3 h 20 min
End Repair	10 min	30 min		20 min		20 min
cDNA clean-up	1 h	1 h		1 h		1 h 30 min
Volume reduction	15 min	45 min		—		—
*In vitro* transcription	25 min	14 h 15 min		14 h		14 h
aRNA clean-up	1 h	1 h		1 h		1 h 30 min
Adapter ligation	20 min	1 h 20 min	1 h	2 h 10 min	1 h	2 h 10 min
Reaction clean up	1 h	1 h		1 h		1 h 30 min
cDNA synthesis	20 min	1 h 20 min		1 h		1 h 10 min
cDNA clean-up	1 h	1 h		1 h		1 h 30 min
Total	6 h	25 h 20 min	2* h	24 h 40 min	2* h	27 h

Hands on time include preparing the reagents and setting up the robot. Total time include reaction times and necessary setups that cannot be performed during a previous incubation step.

^*^Could be shortened to 20 min by using pre-aliquoted reagent plates.
