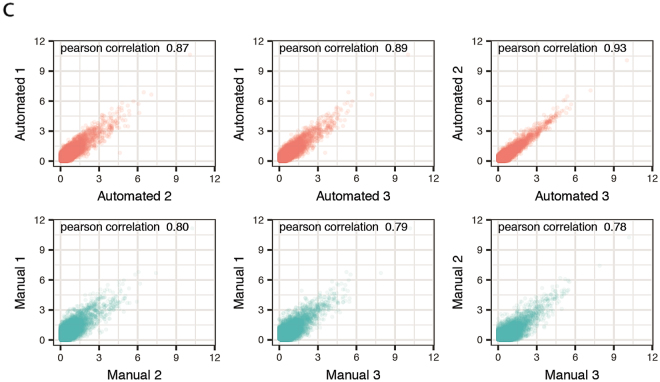# Corrigendum: An automated approach to prepare tissue-derived spatially barcoded RNA-sequencing libraries

**DOI:** 10.1038/srep41109

**Published:** 2017-03-13

**Authors:** Anders Jemt, Fredrik Salmén, Anna Lundmark, Annelie Mollbrink, José Fernández Navarro, Patrik L. Ståhl, Tülay Yucel-Lindberg, Joakim Lundeberg

Scientific Reports 6: Article number: 3713710.1038/srep37137; published online: 11 16 2016; updated: 03 13 2017

This Article contains errors in Figure 6c. The Pearson correlation values for the automated and manually prepared samples were inadvertently switched. The correct Figure 6c appears below as Figure 1.

## Figures and Tables

**Figure 1:**