# Supplementary material for: An automated approach to prepare tissue-derived spatially barcoded RNA-sequencing libraries
Source: Sci Rep. 2016 Nov 16;6:37137. doi: 10.1038/srep37137 (PMC5111054; doi:10.1038/srep37137)
Supplement: Supplementary Information [file srep37137-s1.pdf]

An automated approach to prepare tissue-derived spatially barcoded RNA-sequencing libraries

Jemt A[1], Salmén F[1], Lundmark A[2], Mollbrink A[1], Fernández Navarro J[1], Ståhl PL[3], Yucel-Lindberg T[2] & Lundeberg J[1]*

[1]Science for Life Laboratory, Department of Gene Technology, Royal Institute of Technology, SE-106 91, Stockholm, Sweden

[2]Department of Dental Medicine, Division of Periodontology, Karolinska Institute, SE-141 04, Huddinge, Sweden

[3]Department of Cell and Molecular Biology, Karolinska Institute, SE-171 77 Stockholm, Sweden

*Corresponding author, email: joakim.lundeberg@scilifelab.se

Installing and running the scripts

Magnatrix 8000+ users

The protocol group file, including all scripts necessary to run the protocols has been packaged as a .Hxp file. It can be imported using the import function of the Magnatrix operating system. To import the protocol file, log in to the instrument with an Administrator or Power user account and start the Import Guide from the Tools menu. Follow the prompts on the screen and import the protocols to an AMF file of your choice. Restart the Magnatrix operating system and the new set of protocols should with all the scripts should appear in the chosen AMF file.

The imported Protocol Group consists of two programs to run the protocol in either 1-8 sample mode or 9-16 sample mode, each divided in two parts. The protocols intended to be used with the default settings, but it is possible to tweak settings such as incubation temperatures and incubation times. The protocol uses two Abgene 0600 PCR plates (ThermoFischer Scientific) and one Abgene 2.2 mL Storage plate.

Some small adjustments might be necessary to tailor the script to suit the conditions at other laboratories. The plate with enzymatic reagents are kept cool on one of the Peltier elements and, depending on the ambient temperature and humidity, there is a risk of condensation. It might be necessary to change the cooling temperature to prevent this (stored in the variable EnzymeTemp in the Variable files for each program). The script uses mainly relative movements, which should not require any modifications. However, when brushing the tips against the side of the liquid waste container the movement command is given in absolute coordinates, which might require adjustments. The coordinate is stored in the variable BrushOffPosition in the Varaible file for each script. In order to check if the value needs to be adjusted, add a liquid waste container to the deck in the position indicated by the Configuration file. Attach tips to the head unit and move the head to the liquid waste container using the Positioner tool. Remember to specify in the Positioner that the head has tips attached and that the position contains a Drain reservoir. Move the head stepwise along the Y-axis until the tips brushes against the side of the upper container. Note the Y-axis

coordinate and replace the coordinate stored in the variable BrushOffPosition located in the Variable files with the new coordinate.

Using other liquid handling robots

There are no programs that will automatically convert the Magnatrix scripts for use on other liquid handling robots. However, re-writing the scripts to run on another open liquid handling platform should not be too laborious. Several programs originally written for the Magnatrix system have been adapted to run on a Bravo Automated Liquid Handling Platform (Agilent)[1-3]. Each program consists of five text files that can be viewed and edited in a simple text editor. The Configuration (*.Cfg) file contains the layout of the work deck, e.g. the plates and their position on the work deck together with aliases. The Variable (*.Var) file holds most of the scripts variables used in the specific program. The Prologue (*.Plg) takes the input from the Variable file and calculates such things as volumes and incubation times that are to be used when loading plates and in the script. The Script file (*.Prt) contains the actual movements of the pipetting head unit, temperature settings and incubation control. Finally the General file (*.Gen) holds constant values such as movement speeds in variables that can be called upon in the Script file. Using the information from these files the program can be converted to run on other systems.

Some general points to take into consideration when re-writing the script for other platforms. The platform must be able to perform magnetic separation of beads for the purification steps. As the protocol consists of several enzymatic steps during the run it, is preferable to use a robotic workstation capable of keeping the reagents cool in one position while having another temperature-controlled unit for the enzymatic reactions. Reactions that are incubations at elevated temperatures are covered with oil to prevent evaporation losses. A system that has the capacity to seal plates can obviously omit oil and seal the plates instead. The aspiration volumes in the script have been adjusted depending on the temperature of the aspirated liquid in order to ensure that the correct volume is drawn and will need to be adjusted for a new liquid handler.

Supplementary Tables

Supplementary Table 1: Summary of sequencing results

Sample	Raw read pairs	Processed read pairs	Reads after trimming	Mapped reads	Annotated reads	Unique transcripts
**Auto 1**	170,947,779	170,947,779	143,539,709	125,651,337	53,515,124	4,730,332
**Auto 2**	197,986,936	170,947,779	143,159,827	127,991,172	66,399,975	4,544,269
**Auto 3**	175,345,901	170,947,779	143,322,576	123,793,582	59,842,770	3,860,600
**Man 1**	205,230,475	170,947,779	118,687,184	93,405,521	40,723,886	3,701,146
**Man 2**	218,123,282	170,947,779	121,827,981	99,105,585	46,002,490	3,591,339
**Man 3**	195,356,551	170,947,779	141,175,314	124,141,868	47,876,628	2,886,587

Supplementary Figures

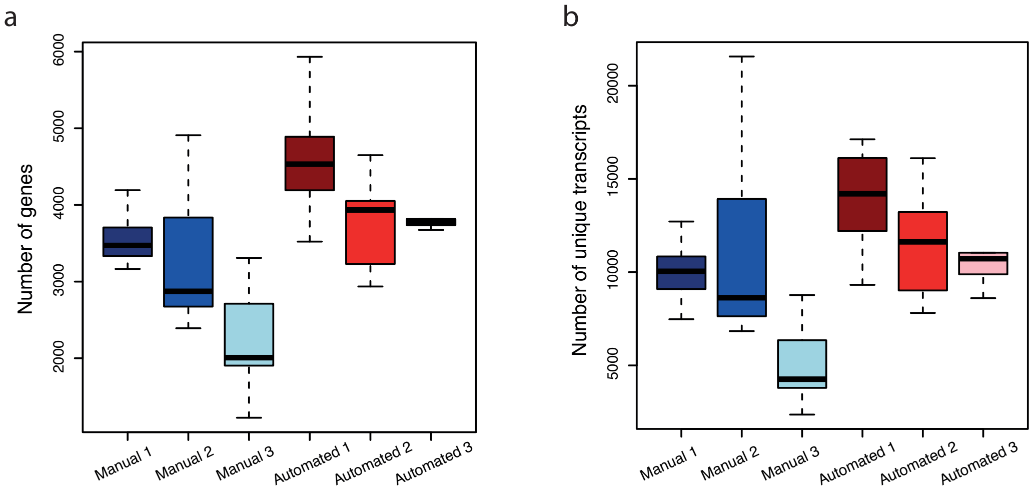

Supplementary figure 1: Boxplots of the number of genes (a) and unique transcripts (b) detected in the selected inflamed regions.

The boxplots are separated on library type where the three leftmost boxplots in each figure panel corresponds to the three manual replicates while the data for three rightmost originates from the automated replicates. Each boxplot displays the variation in the number of genes (a) or unique transcripts (b) among the ten spots aligning directly under the inflamed region.

References

1 Lundin, S., Stranneheim, H., Pettersson, E., Klevebring, D. & Lundeberg, J. Increased throughput by parallelization of library preparation for massive sequencing. *PloS one* **5**, e10029-e10029, doi:10.1371/journal.pone.0010029 (2010).

2 Stranneheim, H., Werne, B., Sherwood, E. & Lundeberg, J. Scalable transcriptome preparation for massive parallel sequencing. *PloS one* **6**, e21910-e21910, doi:10.1371/journal.pone.0021910 (2011).

3 Borgström, E., Lundin, S. & Lundeberg, J. Large scale library generation for high throughput sequencing. *PloS one* **6**, e19119-e19119, doi:10.1371/journal.pone.0019119 (2011).
